# Chloroplast Electron Chain, ROS Production, and Redox Homeostasis Are Modulated by COS-OGA Elicitation in Tomato (*Solanum lycopersicum*) Leaves

**DOI:** 10.3389/fpls.2020.597589

**Published:** 2020-12-14

**Authors:** Sophie Moreau, Géraldine van Aubel, Rekin’s Janky, Pierre Van Cutsem

**Affiliations:** ^1^Research Unit in Plant Cellular and Molecular Biology, Biology Department, Institute of Life, Earth and Environment, University of Namur, Namur, Belgium; ^2^FytoFend S.A., Isnes, Belgium; ^3^VIB Nucleomics Core, Leuven, Belgium

**Keywords:** chloroplast, acclimation, photosynthesis, plant immunity, redox homeostasis, ROS, elicitation

## Abstract

The stimulation of plant innate immunity by elicitors is an emerging technique in agriculture that contributes more and more to residue-free crop protection. Here, we used RNA-sequencing to study gene transcription in tomato leaves treated three times with the chitooligosaccharides–oligogalacturonides (COS-OGA) elicitor FytoSave^®^ that induces plants to fend off against biotrophic pathogens. Results showed a clear upregulation of sequences that code for chloroplast proteins of the electron transport chain, especially Photosystem I (PSI) and ferredoxin. Concomitantly, stomatal conductance decreased by half, reduced nicotinamide adenine dinucleotide phosphate [NAD(P)H] content and reactive oxygen species production doubled, but fresh and dry weights were unaffected. Chlorophyll, β-carotene, violaxanthin, and neoxanthin contents decreased consistently upon repeated elicitations. Fluorescence measurements indicated a transient decrease of the effective PSII quantum yield and a non-photochemical quenching increase but only after the first spraying. Taken together, this suggests that plant defense induction by COS-OGA induces a long-term acclimation mechanism and increases the role of the electron transport chain of the chloroplast to supply electrons needed to mount defenses targeted to the apoplast without compromising biomass accumulation.

## Introduction

In their natural environment, plants are parasitized by a vast array of pathogens and other pests, including viruses, fungi, bacteria, and insects. Upon pest attack, several metabolic pathways are activated to strengthen external barriers, set up an epigenetic control of defense, and develop a toxic environment around the pest (Ramirez-Prado et al., 2018).

To trigger such defense responses, the biotic threat has to be detected. This initial recognition is based on chemical cues released by and characteristic of the aggressor, referred to as microbe- or pathogen-associated molecular patterns (MAMPs or PAMPs, respectively; Gust et al., 2017). Following infection or injury, the host plant releases molecules called damage-associated molecular patterns (DAMPs). MAMPs, PAMPs, and DAMPs lead to the development of the PAMP-triggered immunity (PTI) through recognition by pattern recognition receptors (PRRs) mainly located in the plant plasma membrane (Couto and Zipfel, 2016; Gust et al., 2017; Jamieson et al., 2018). This primary innate immunity may be suppressed by effector proteins produced by pathogens. These effectors trigger a specific defense reaction called effector-triggered immunity (ETI). However, the PTI-ETI dichotomy weakly reflects the complexity of the host defense system. Recently, the “invasion model” has been proposed as an alternative concept. This model describes a global surveillance system in which any molecule may potentially be detected as an invasion pattern (IP; Cook et al., 2015; Kanyuka and Rudd, 2019).

A similar defensive response can be achieved by spraying compounds, called elicitors or resistance inducers, which are used as a preventive treatment. The “chitooligosaccharides–oligogalacturonides (COS-OGA)” elicitor combines plant non-self-molecules, i.e., chitosan-derived chitooligosaccharides (COS), and plant self-compounds, i.e., pectin-derived oligogalacturonides (OGA; Cabrera et al., 2010). This elicitor has already proven its effectiveness against powdery mildew on tomato, grapevine, and cucumber, a disease caused by the biotrophic pathogens *Leveillula taurica*, *Erysiphe necator*, and *Sphaerotheca fuliginea*, respectively, (van Aubel et al., 2014, 2016). The COS-OGA mode of action probably relies on systemic acquired resistance (SAR; Choi et al., 2016). In *Arabidopsis thaliana* cell suspension, it induces apoplast alkalinization and potassium efflux (Cabrera et al., 2010; Liang and Zhou, 2018; Marcec et al., 2019). In tomato and potato, multiple COS-OGA sprayings activate a defense response in a cumulative process that involves salicylic acid, cell wall peroxidases, and PR-protein production (van Aubel et al., 2016, 2018; Clinckemaillie et al., 2017; van Butselaar and Van den Ackerveken, 2020). In rice, COS-OGA triggers a systemic defense response against the root-knot nematodes (*Meloidogyne graminicola*) that does not rely on salicylic acid or jasmonic acid but rather on the phenylpropanoid pathway (Singh et al., 2019). However, the effect of multiple COS-OGA sprayings on plant metabolism has not been investigated much, especially regarding the chloroplast and photosynthesis.

More and more studies highlight the involvement of chloroplast in plant immunity, as evidenced by the large amount of effectors that target this organelle (Lu and Yao, 2018; Kretschmer et al., 2020). Being the site of production of phytohormones, reactive oxygen species (ROS) and secondary metabolites, chloroplasts play a major role in plant response to biotic stresses: chloroplasts serve as a hub in biotic and abiotic stress responses to enable physiological adjustment following stimulus perception (Mühlenbock et al., 2008; Sano et al., 2014; Stael et al., 2015; Serrano et al., 2016; Lu and Yao, 2018).

A direct link between PAMP detection and the chloroplast has already been identified. Flg22 treatment induces the translocation of the plasma membrane-localized protein CPK16 and triggers the expression of defense-related genes in the nucleus (Medina-Puche et al., 2020). Calcium constitutes a key element for chloroplast response, and both abiotic and biotic stresses including chitin, flg22, cold, and NaCl induce a Ca^2+^ rise in the stroma (Stael, 2019; Teardo et al., 2019; Navazio et al., 2020). Numerous other signal transducers have been proposed including the calcium-sensing (CaS) protein, mitogen-activated protein kinase (MAPK) cascades, ROS, tetrapyrroles, intermediate products of the isoprenoid biosynthesis, and carotenoid derivatives (Leister, 2017; Teardo et al., 2019; Wu et al., 2019; Jiang et al., 2020; Wang et al., 2020a). Finally, this retrograde signaling modulates the expression of (a)biotic stress- and photosynthesis-related genes to adapt the photosynthetic machinery (Wang et al., 2020b). It is indeed generally thought that the activation of plant immune response induces plant growth reduction and a decrease in photosynthesis (Bilgin et al., 2010; Baccelli et al., 2020). Nonetheless, several articles using benzothiadiazole (BTH), chitosan, or the commercial product Romeo^®^ containing cell walls of *Saccharomyces cerevisiae* describe an upregulation of photosynthesis-related genes suggested to be linked to ROS production, an increased demand in energy for defense responses, and/or an increased photosynthetic efficiency (Landi et al., 2017; De Miccolis Angelini et al., 2019; Lemke et al., 2020).

Chloroplasts maintain energy and redox homeostasis by adjusting photochemistry and the Calvin–Benson cycle according to environmental stress sensing. In particular, high light intensity and stomatal closure both lead to excess energy in photosystems that has to be dissipated to avoid photooxidative damages. Dissipating mechanisms include non-photochemical quenching (NPQ) that dissipates energy through heat. NPQ regroups different components: the zeaxanthin-dependent (qE), the state transition based on LHCII migration between the Photosystem II (PSII) and Photosystem I (PSI; qT) and the photoinhibitory quenching (qI; Göhre et al., 2012; Foyer, 2018). In addition to NPQ, other acclimation mechanisms enable the plant to adapt to adverse conditions including the cyclic electron flow (CEF), the adjustment in the photosynthetic apparatus, and ROS scavenging systems (Schöttler and Tóth, 2014; Sunil et al., 2019; Bielczynski et al., 2020). These adaptations in response to abiotic stresses are induced systemically, a phenomenon referred to as systemic acquired acclimation (SAA; Morales and Kaiser, 2020). Interestingly, chitosan treatment leads to a decrease in NPQ, which may reflect an improved efficiency of light utilization (Qu et al., 2019; Mukhtar Ahmed et al., 2020). Chitosan-based elicitors also seem to alleviate the detrimental effects of abiotic stresses (e.g., drought, cadmium, and salinity) on photosynthesis, growth, and yield (Ghasemi Pirbalouti et al., 2017; Zayed et al., 2017; Alkahtani et al., 2020; Li et al., 2020).

Only a handful of articles focuses on interactions between elicitation and photosynthesis. To investigate the plant response to COS-OGA elicitation, we assessed the transcriptional reprogramming [RNA-sequencing (RNA-Seq)] of tomato plants after repeated leaf sprayings with this elicitor. The results of this RNA-Seq prompted us to further investigate the photosynthesis response and ROS production following COS-OGA spraying.

## Materials and Methods

### Plant Material and Treatment

Tomato plants (*Solanum lycopersicum* cv. Moneymaker) were grown on loam at 24°C with a 16-h/8-h day/night regime and a light intensity of 600–700 μmol m^–2^ s^–1^ (white fluorescent lamp). 3 weeks after sowing, plants were sprayed three times each with 16 ml of either 0.5% (v/v) FytoSave^®^ (12.5 g/l COS-OGA, FytoFend, Belgium) or 0.1% Tween 20 (control) at 7, 3, and 1 day before harvest. Control plants were sprayed with 0.1% Tween 20 to exclude any specific effect of the surfactant contained in the FytoSave^®^. The day before each treatment, plants were transferred into a growth cabinet (GC-1000, Lab Companion, South Korea) maintained on a 16-h/8-h day/night photoperiod at 20°C and 90% relative humidity (RH).

For RNA-Seq, pigment content, reduced nicotinamide adenine dinucleotide phosphate [NAD(P)H] content, and peroxidase activity, plant leaves were sampled in liquid nitrogen. The samples were ground with a mixer mill MM 400 (Retsch, Germany) using sterile grinding jars cooled in liquid nitrogen and stored at −80°C.

### RNA Extraction and RNA-Sequencing

Plant leaves from six biological replicates were harvested 24 h after the third spraying. RNA extraction was performed on 100 mg ground nitrogen-frozen leaves using NucleoSpin^®^ RNA Plant (Macherey-Nagel, Germany). RNA concentration, purity, and integrity were assessed with Bioanalyzer 2100 (Agilent, United States).

Library preparation was made with TruSeq Stranded mRNA kit (Illumina, United States) on 1 μg total RNA. Libraries were then sequenced on NextSeq 500 Platform (Illumina, United States) following the standard protocol (75 bp single-end reads, High-Output kit) at the VIB Nucleomics core^1^.

Low-quality ends and adapter sequences were trimmed off from the Illumina reads with FastX 0.0.14^2^ and Cutadapt 1.15 (Martin, 2011). Subsequently, small reads (length < 35 bp), polyA-reads (more than 90% of the bases equal A), ambiguous reads (containing N), low-quality reads (more than 50% of the bases < Q25), and artifact reads (all but three bases in the read equal one base type) were filtered using FastX 0.0.14 and ShortRead 1.36.0 (Morgan et al., 2009; Martin, 2011). With Bowtie2 2.3.3.1 (Langmead and Salzberg, 2012), we identified and removed reads that align to Phix Control (Illumina, United States).

Reads were aligned by STAR 2.5.2b to the reference genome (*S. lycopersicum* SL2.50.38; Dobin et al., 2013). Default parameters were used, except for two pass Mode (Basic). Using Samtools 1.5, reads with a mapping quality smaller than 20 were removed from the alignments (Li et al., 2009). Cufflinks v2.2.1 and mergeBed from the Bedtools toolkit were used to reconstruct transcripts and to identify features using reference annotation (Quinlan and Hall, 2010; Trapnell et al., 2010).

Read counts for each gene were obtained with featureCounts 1.5.3. Reads that could be attributed to more than one gene or could not be attributed to any gene were not taken into account (Robinson and Smyth, 2007; Risso et al., 2011; Liao et al., 2014). The next steps of the analysis were conducted using R (R-3.5.3) with Bioconductor package. EDASeq was used to normalize each sample using full quantile normalization according to its GC content, library size, and RNA composition. Based on Spearman correlation, hierarchical clustering highlighted an outlier in tomato plants, which was therefore removed from the analysis.

Differentially expressed genes (DEGs) were identified with edgeR 3.20.8 based on a negative binomial generalized linear model (GLM). We did not use the normalized counts directly but worked with offsets. False discovery rates (FDRs) were calculated based on *p*-values with Benjamini–Hochberg method to correct for multiple comparison errors. DEGs were defined as presenting an FDR value < 0.05 and an absolute log_2_ Fold change larger than one (${\text{log}}_{2}\frac{\text{COS}-\text{OGA}}{\text{Control}}$, further referred as log_2_ FC). The sequence data were submitted to NCBI Sequence Read Archive database (https://www.ncbi.nlm.nih.gov/sra, Accession number: PRJNA645061).

### MapMan Visualization and Functional Analysis

Differentially expressed genes were visualized in metabolic pathways using MapMan Software (version 3.6) and the Slyc_ITAG2.3 Mapping file. Gene classification in MapMan bins was compared to functional information found on Plaza platform (Dicot 4.5, VIB; Van Bel et al., 2018). The integrative orthology viewer (Dicot 4.5, VIB) was used to search for orthologs and paralogs when needed. The newly created mapping file is given in Supplementary Table 1. A custom diagram was finally produced to include different biological pathways.

Upregulated DEGS were also assigned to Gene Ontology (GO) categories (biological pathways and cellular components) using Plaza platform (Dicot 4.5, VIB; Van Bel et al., 2018). Significantly enriched functions were identified based on a hypergeometric test with Bonferroni correction (*p* values < 0.001). The overrepresented and/or depleted GO terms were characterized by an enrichment fold corresponding to their frequency in the gene set in comparison to their genome-wide background frequency (protein coding genes with or without annotations).

### Chlorophyll Fluorescence Parameters

Photosynthesis parameters were assessed on plants sprayed one and three times each with 16 ml of either COS-OGA (62.5 mg/l) or 0.1% Tween 20 on both sides of the leaves. Directly after spraying, plants were dark-adapted during 20 min. Thereafter, fluorescence was measured *in vivo* with a closed FluorCam (FluorCam 800 MF, Photon Systems Instruments, Czech Republic) and the associated software with the program Quenching Act1 in Pulse-Amplitude modulated mode (PAM).

Briefly, the program starts with a dark phase characterized by minimal fluorescence emissions in dark-adapted plants (F_0_). The maximum fluorescence in dark-adapted plants (F_M_) is then measured by photosystem saturation with a strong light flash. F_M_ and F_0_ enable the calculation of the maximum quantum yield in the dark-adapted state (F_v_/F_M_). The program continues with an actinic phase (red light) interrupted by five saturation flashes enabling the measurement of steady-state fluorescence emissions in light (F_t__Lss), the minimal fluorescence emissions in light-adapted plants (F_0__Lss), and the maximal fluorescence emissions in light-adapted plants (F_M__Lss). These data were used to determine NPQ, the fraction of oxidized PSII reaction centers (qL), and PSII operating efficiency (ΦPSII). These measurements were reassessed on other plants 24 h after the first and the third sprayings. All parameters were assessed on six biological replicates. Parameters were calculated as follows:

$$F_{V}/F_{M}=\frac{F_{M}-F_{0}}{F_{M}}$$

$$NPQ=\frac{F_{M}-F_{M}\_Lss}{F_{M}\_Lss}$$

$$qL=\frac{\left(F_{M}\_Lss-F_{t}\_Lss\right)/\left(F_{M}\_Lss-F_{0}\_Lss\right)}{F_{0}\_Lss/F_{t}\_Lss}$$

$$\Phi PSII=\frac{F_{M}\_Lss-F_{t}\_Lss}{F_{M}\_Lss}$$

### Pigment Content

Pigment extraction was performed as previously described (Hu et al., 2013). Briefly, tomato leaves were sampled in liquid nitrogen 24 h after the third spraying. Ground frozen leaves (0.1 *g*) were incubated in 1 ml 100% acetone at −20°C for 24 h in the dark. Samples were centrifuged at 17,000 *g* for 10 min at 4°C. The resulting supernatant (50 μl) was analyzed by high-performance liquid chromatography (HPLC) with a C18 column (MN C18 Nucleodur^®^ 100-5, 125 mm × 4 mm, Macherey-Nagel, Germany) as reported previously (Wright, 1991). The absorbance was measured at 430, 440, 450, and 460 nm. The peak area was integrated at 430 nm for chlorophyll *a*, 440 nm for neoxanthin and violaxanthin, 450 nm for β-carotene, and 460 nm for chlorophyll *b*. Each peak was compared to its respective standard (DHI Laboratory Product, Denmark) to assess the pigment content in elicited and control leaves. Results are given as mg per g (fresh weight) ± standard deviation of 12 biological replicates. In another experiment, tomato leaves were sampled in liquid nitrogen just before the third spraying (i.e., 2 days after the second spraying) and 24 h after this third spaying. The same protocol was used to measure chlorophyll *a* and *b* content. Average pigment contents were expressed as percentages of the control ± standard deviation from six biological replicates.

### Stomatal Conductance

Stomatal conductance was measured 24 h after the third spraying using AP4 leaf porometer (Delta-T Devices, United Kingdom) on the lower surface of the third true leaf (24°C and 60% RH). Results were expressed in percentage of the control ± standard deviation of 12 biological replicates.

### Fresh and Dry Weight of Aerial Parts

Both fresh and dry (60°C for 3 days) weights were determined from the aerial parts of the plants 24 h and 7 days after the third elicitation. Results were expressed in percentage of the control ± standard deviation of 10 biological replicates.

### Reduced Nicotinamide Adenine Dinucleotide Phosphate Content

To evaluate NAD(P)H content 24 h after the third spraying, 0.1 *g* ground frozen leaves were homogenized with an Ultra Turrax (IKA, Germany) in 500 μl phosphate buffer (50 mM and pH 7.4). Samples were then centrifuged at 10,000 *g* for 10 min at 4°C. The supernatant was kept and centrifuged at 4°C for 10 min at 13,000 *g*. The resulting supernatants were filtered using 10-kDa molecular weight cutoff (MWCO) Spin Filter (Sigma-Aldrich, United States). NAD(P)H content was evaluated using NAD(P)H-Glo Detection System (Promega, United States) on 50-μl filtrates. Average NAD(P)H contents were expressed as a percentage of the controls ± standard deviation from six biological replicates.

### Protein Extraction and Guaiacol Peroxidase Assay

Tomato leaves were sampled in liquid nitrogen 24 h after the third spraying. For peroxidase assays, 0.5 *g* ground whole frozen leaves were homogenized with an Ultra Turrax (IKA, Germany) in 2 ml extraction buffer [50 mM sodium acetate, 1 M NaCl, 5 mM ethylenediaminetetraacetic acid (EDTA), and pH 5.2]. The samples were centrifuged for 10 min at 17,000 *g* and 4°C. The supernatant was kept and centrifuged at 4°C for 10 min at 13,000 *g*. The resulting protein extracts (40 μl) were mixed with 975 μl of 180 mM guaiacol (Sigma-Aldrich, United States). Then, 10 μl H_2_O_2_ were added to 250 μl of each extract, and guaiacol peroxidase activity was measured for 5 min at 420 nm (Multiskan Go spectrophotometer, Thermo Fisher Scientific, United States).

The total protein contents were determined using Pierce 660 nm Protein Assay (Thermo Fisher Scientific, United States) and used to normalize the peroxidase activities. All average guaiacol peroxidase activities were then expressed as a percentage of the control (*n* = 12).

### Apoplastic Reactive Oxygen Species Production

Apoplastic ROS were measured using GloMax-Multi + Detection system (Promega, United States) using a protocol modified from an earlier described protocol (Bisceglia et al., 2015). Briefly, biopsy punches (4 mm diameter, Kai Medical, Japan) were used to collect four leaf disks per plant from the mesophyll of the second true leaf. These samples were collected from six 4-week-old tomato plants that had not been previously sprayed. The collected leaf disks were placed in a Petri dish containing distilled water for 2 h in the dark. Every 30 min, the distilled water was replaced to remove cellular damage-related compounds. Leaf disks were placed in 96-well microplates (Lumitrac 200, Greiner Bio-One, United States) with 100 μl MS medium (4.43 g/l MS pH 5.7, Duchefa Biochemie, Netherlands) and 50 μl luminol/peroxidase solution containing 9 μg/ml luminol and 4 μg/ml peroxidase (Sigma-Aldrich, United States). After 4 h incubation at 24°C, 50 μl of 62.5 mg/l COS-OGA for elicited leaf disks or 50 μl 0.1% Tween 20 for control leaf disks were added. The chemiluminescence was measured directly for 90 min with a signal integration time of 1 s. Each curve was integrated and expressed in relative chemiluminescence units. The average value of chemiluminescence per treatment was expressed in percentage of the control (*n* = 12).

### Statistical Analysis

Statistical analyses were conducted using R (R-3.5.3). The normality of residuals was checked using a Shapiro–Wilk normality test. When data were normally distributed, they were compared using a Student’s *t* test. The non-parametric Wilcoxon signed-rank test was used for non-normally distributed data.

## Results

### RNA Sequencing

RNA-Seq data were generated from tomato plants sprayed three times with COS-OGA (62.5 mg/l) or 0.1% Tween 20 and harvested 24 h after the third treatment. More than 384 million preprocessed reads of 75 pb were obtained after sequencing with an average 16 million reads per sample. After quality filtering, 95.7% of processed reads were mapped against tomato genome (*S. lycopersicum* SL2.50.38). The proportion of mapped reads that were finally taken into account for expression levels reached 89.1% (13 million reads/sample on average).

Genes with | log_2_ FC | greater than 1 and an FDR less than 0.05 were considered as differentially expressed (DEGs). Following COS-OGA application, 849 genes were differentially expressed, in which 496 were upregulated and 353 downregulated. Among these genes, 799 were considered as protein coding genes (447 upregulated and 352 downregulated). These protein coding genes were used for MapMan visualization and the enrichment analysis.

### MapMan Visualization

**FIGURE 1:**
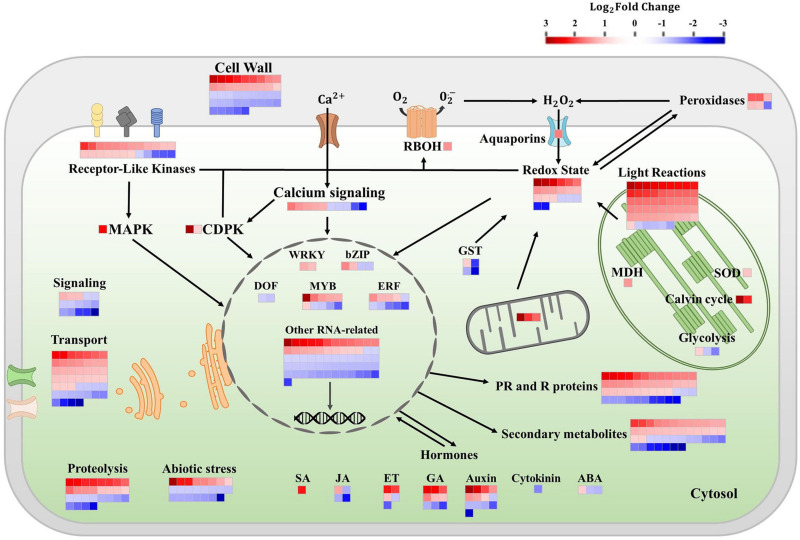
Differentially expressed genes from selected pathways in tomato leaves after three sprayings with chitooligosaccharides–oligogalacturonides (COS-OGA; 62.5 mg/l; based on MapMan). Tomato leaves were harvested 24 h after the third treatment. Represented genes show a significant change in expression [false discovery rate (FDR) < 0.05; |log_2_Fold Change| > 1]. Each square corresponds to log_2_ fold change of a transcript in elicited versus control samples. Squares are colored in red and blue if they are classified as upregulated and downregulated, respectively. The names of the genes and their relative expression levels are given in Supplementary Table 1. ABA, abscisic acid; bZip, basic leucine zipper domain; CDPK, calcium-dependent protein kinase; DOF, DNA-binding with one finger; ERFs, ethylene responsive factors; ET ethylene; GA, gibberellic acid; GST, glutathione-S-transferase; JA, jasmonic acid; MAPK, mitogen-activated protein kinase; MDH, NADP malate dehydrogenase; PR, pathogenesis-related; R, resistance; RBOH, respiratory burst oxidase; SA, salicylic acid; and SOD, superoxide dismutase.

Transcripts of DEGs were visualized in metabolic pathways using MapMan software (version 3.6). First of all, 82.0% of the data set were successfully attributed to a specific functional category (i.e., bin), and the remaining entries were classified as “not assigned” (146 genes, 66 upregulated, and 80 downregulated). The complete set of genes is given in Supplementary Table 2. The main functional categories influenced by COS-OGA spraying are summarized in Figure 1. These pathways correspond to hormonal signaling, photosynthesis, signal transduction (including receptor-like kinases, calcium signaling, and MAPKs), transcription factors, transport, abiotic stress, proteolysis, cell wall, redox homeostasis, mitochondrion, pathogenesis-related and resistance proteins (PR and R proteins), and secondary metabolites.

Following three COS-OGA sprayings, 51 transcripts related to light reactions were regulated (46 upregulated and five downregulated). These transcripts belong to PSII (20 upregulated and one downregulated), PSI (23 upregulated and one downregulated), ferredoxin (log_2_ FC = 2.89), cytochrome b6/f (log_2_ FC = 1.47 and 1.03), and chlorophyll biosynthesis (three genes downregulated). In PSII, 20 genes corresponding to chlorophyll apoprotein CP43 and CP47, reaction center protein H and Z, Q(B) protein, and D2 protein were upregulated (log_2_ FC between 1.18 and 1.89), while the chloroplastic protein HCF243 was downregulated (log_2_ FC = −1.43). Concerning PSI, 21 transcripts related to chlorophyll *a* apoprotein (A1 and A2), one protein ycf4, and one NDH gene involved in CEF were upregulated in elicited tomatoes (log_2_ FC from 1.43 to 2.73), while one transcript of the reaction center subunit IV A was downregulated (log_2_ FC = −1.09). Chlorophyll biosynthesis-related genes were repressed: two of these genes were protochlorophyllide reductases (log_2_ FC = −1.24 and −1.18) and another one was chlorophyllase (log_2_ FC = −1.27). Two genes belonging to the Calvin–Benson cycle were also upregulated, namely, chloroplastic carbonic anhydrase (log_2_ FC = 3.07), and Rubisco (log_2_ FC = 2.24). Other transcripts associated with the chloroplast were influenced by COS-OGA treatments such as glycolysis- and/or gluconeogenesis-related proteins: pyruvate kinase (log_2_ FC = 1.03), fructose-bisphosphate aldolase (log_2_ FC = −1.15), and fructose-1,6-bisphosphatase (log_2_ FC = −1.70). An NADP malate dehydrogenase (MDH, log_2_ FC = 1.55) implicated in the malate valve and responsible for the interconversion between malate and oxaloacetate (OAA) was also upregulated. Concerning mitochondrial electron transport, three genes were upregulated: an ATP synthase (log_2_ FC = 3.50), a cytochrome b (log_2_ FC = 2.17), and a cytochrome c oxidase (log_2_ FC = 1.87).

Genes associated with redox homeostasis were mainly upregulated (23 upregulated and nine downregulated). Redox-related DEGs include six cell wall peroxidases (five with log_2_ FC between 1.17 and 1.92, one log_2_ FC = −1.80), four glutathione S-transferases (log_2_ FC = −2.67, −2.15, −1.37, and 1.06), one respiratory burst oxidase (RBOH; log_2_ FC = 1.58), one plastidial superoxide dismutase (log_2_ FC = 1.16), three genes involved in the thioredoxin system (log_2_ FC = 2.01, 1.03, and −1.06), one catalase (log_2_ FC = −1.05), one glutaredoxin (log_2_ FC = −2.29), one nucleoredoxin (log_2_ FC = 1.25), and 14 other genes classified as involved in redox homeostasis or with an oxidoreductase activity (12 upregulated with log_2_ FC between 1.05 and 3.37, two downregulated with log_2_ FC = −1.09 and −2.34).

In signal transduction pathways, 52 transcripts were regulated. Receptor-like kinase transcripts (RLKs, 24 transcripts), MAPK (log_2_ FC = 2.46), and calcium-dependent protein kinases (CDPKS, log_2_ FC = 3.76 and 1.00) were largely upregulated. Half of RLK genes were assigned to leucine-rich repeat receptors (LRRs), both upregulated and downregulated (log_2_ FC ranging between −2.00 and 2.01). All the other receptor types were upregulated and corresponded to S-receptor kinases, cysteine-rich receptor-like kinases (CRKs), wall-associated kinase (WAK), and unspecified receptor kinases (log_2_ FC from 1.03 to 2.20). Calcium signaling was both upregulated and downregulated (10 transcripts, log_2_ FC between 1.76 and −2.40). The other gene classes involved in signal transduction (phosphoinositide, sugar and nutrient signaling, vesicle, G-proteins, and light signaling) were mainly downregulated.

Besides, 41 transcripts had a function related to the cell wall. The most regulated genes were involved in cell wall modifications by acting on hemicellulose: seven xyloglucan endotransglucosylase/hydrolases (XTH) were upregulated (log_2_ FC from 1.37 to 2.81), and only one was downregulated (log_2_ FC = −1.48). Two other hemicellulose-related transcripts were downregulated: a mannan endo-1,4-beta-mannosidase (log_2_ FC = −1.05) and a beta-xylosidase (log_2_ FC = −1.21). Concerning cellulose, five cellulose synthase transcripts and an endo-1,4-beta-glucanase were downregulated (log_2_ FC from −1.03 to −1.55), and a Cobra-Like protein involved in the orientation of cellulose microfibrils was upregulated (log_2_ FC = 2.05). Thirteen regulated transcripts were associated with pectin: four polygalacturonases (log_2_ FC = 1.39, −1.04, −1.16, and −1.19), five pectinesterase/pectinesterase inhibitors (log_2_ FC = 1.48, 1.37, −1.49, −1.68, and −1.89), two rhamnogalacturonate lyases (log_2_ FC = 1.58 and −1.41), one pectate lyase (log_2_ FC = 1.03), and one glycosyltransferase (log_2_ FC = −1.47). Finally, 11 other cell wall-related transcripts were also regulated. Those included genes coding for one extensin (log_2_ FC = 1.57), one cell wall precursor (log_2_ FC = 1.33), three fasciclin-like arabinogalactan proteins (log_2_ FC = −1.29, −1.70, and −2.05), and two proteins involved in secondary cell wall synthesis (log_2_ FC = −1.24 and −1.17).

The most regulated hormonal pathways were auxins (13 transcripts) and gibberellins (nine transcripts), showing both positive and negative effects. One DEG corresponding to an auxin efflux carrier protein was highly upregulated with a log_2_ FC = 3.86. The expression of genes in the jasmonic acid pathway mainly decreased (one upregulated and three downregulated), while a gene associated with the salicylic acid pathway showed an increased expression (log_2_ FC = 2.33). Abscisic acid, ethylene, and cytokinin pathways were upregulated and downregulated concurrently.

An important number of DEGs matched secondary metabolism and PR proteins. PR proteins and R proteins were represented by 46 transcripts and were mainly upregulated (33 upregulated and 13 downregulated). They included chitinases, proteinase inhibitors, PR1, phloem lectins, and R proteins involved in ETI. Concerning the secondary metabolism, 43 DEGs were identified. The main regulated class corresponded to flavonoids (12 transcripts) and simple phenols metabolism (11 transcripts). Flavonoid-related genes were largely upregulated (11 transcripts with a log_2_ FC between 2.11 and 1.12 and one with log_2_ FC = −1.11). Regarding simple phenols, 10 DEGs corresponded to laccase-coding genes with three upregulated and seven downregulated transcripts. Other transcripts related to secondary metabolites were clustered between categories associated with phenylpropanoids (10 DEGs both upregulated and downregulated), alkaloids (five downregulated DEGs with a log_2_ FC between −1.11 and −2.34), wax (log_2_ FC = 1.84 and 1.19), terpenoids (log_2_ FC = 1.08 and −1.15), shikimate (log_2_ FC = 1.39), carotenoids (log_2_ FC = 1.03), and lignans (log_2_ FC = 1.19).

The transport category contained 47 COS-OGA-responsive transcripts. The main class affected by elicitation corresponded to ABC transporters and multidrug resistance systems. Besides, other classes of transport genes were also modulated by COS-OGA, i.e., lipid transfer proteins, plasma membrane aquaporin, tonoplast aquaporin, porin/voltage-dependent channel, proton pump, transporters related to protein import into chloroplast stroma, malate, calcium, potassium, sulfate, phosphate, sugar, peptides, nucleotide and nucleotide sugar, amino acids, and metals.

In the RNA-related categories, 89 genes were regulated by COS-OGA treatment. They included genes associated with biotic stress such as WRKY, bZIP, DOF, MYB, and ethylene response factors (ERFs). Finally, COS-OGA also influenced the transcription of a subset of genes related to abiotic stresses (23 DEGs) and proteolysis (28 DEGs).

### Enrichment Analysis

**FIGURE 2:**
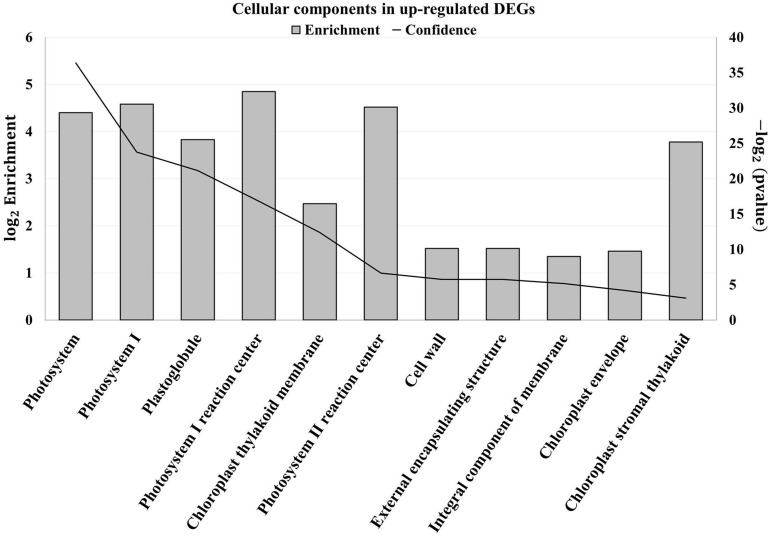
Enriched cellular components (Gene Ontology) in upregulated differentially expressed genes (DEGs) of tomato leaves after three sprayings with chitooligosaccharides–oligogalacturonides (COS-OGA; 62.5 mg/l; based on Plaza project, Dicot 4.5). Tomato leaves were harvested 24 h after the third treatment. Enriched cellular components (Gene Ontology) were identified based on hypergeometric test with Bonferroni correction (*p* values < 0.001). The left axis corresponds to the log_2_ enrichment fold. The right axis represents the −log_2_ (*p* value) represented on the chart by the confidence.

An enrichment analysis was conducted using Plaza platform (Dicot 4.5, VIB) to determine whether some cellular components or biological processes were overrepresented in upregulated DEGs. Concerning the cellular components, 11 GO terms were overrepresented and no depletion was found (Figure 2).

The first cellular component that was clearly overrepresented was the chloroplast with nine GO terms. The terms Photosystem (GO:0009521), PSI (GO:0009522), Plastoglobule (GO:0010287), PSI reaction center (GO:0009538), Chloroplast thylakoid membrane (GO:0009535), PSII reaction center (GO:0009539), and Chloroplast stromal thylakoid (GO:0009533) mainly contain genes related to the light reactions of photosynthesis. The term Chloroplast envelope (GO:0009941) regrouped, among others, genes coding for carbonic anhydrase, superoxide dismutase, MDH, and amino acid transporters.

The apoplast was also overrepresented with two enriched GO terms: the cell wall (GO:0005618) and its parental term “External encapsulating structure” (GO:0030312). These two categories contain the same genes that include XTHs, proteinase inhibitors, laccases, Cobra-like proteins, transporters, and receptor-like kinases. Finally, the term Integral component of membrane (GO:0016021) contains genes coding for transporter and proteins found in thylakoid membranes.

Concerning biological pathways enriched in upregulated DEGs, 12 GO terms were overrepresented, whereas no depletion was found (Supplementary Figure 1). Six terms were related to photosynthesis: Photosynthesis (GO:0015979), Photosynthesis light reactions (GO:0019684), Photosynthesis light harvesting (GO:0009765), Photosynthesis light harvesting in PSI (GO:0009768), Photosynthetic electron transport in PSII (GO:0009772), and Photosynthesis light harvesting in PSII (GO:0009769). The defense response to fungus (GO:0050832) was also enriched in upregulated DEGs with its parental terms Response to fungus (GO:0009620), Defense response (GO:0006952), and Response to stress (GO:0006950). These terms contain genes coding for peroxidases, XTHs, RBOH, CDPKs, proteinase inhibitors, chitinases, PR proteins, RLKs, and calcium signaling. Flavonoid synthesis (GO:0009813) and carbohydrate metabolic processes (GO:0005975) were also enriched, the latter corresponding to chitinases and cell wall-related proteins such as XTHs.

### Chlorophyll Fluorescence Parameters

**FIGURE 3:**
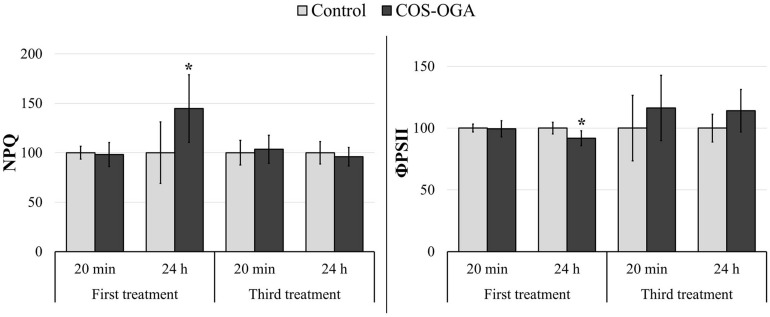
Chlorophyll fluorescence parameters of tomato plants 20 min and 24 h after the first and the third sprayings with chitooligosaccharides–oligogalacturonides (COS-OGA; 62.5 mg/l). PSII operating efficiency (ΦPSII) and non-photochemical quenching (NPQ) were calculated from fluorescence kinetic data generated during the quenching protocol (Fluorcam). These parameters were determined after 20 min dark adaptation. The average values of fluorescence parameters are expressed as a percentage of the control ± standard deviation (*n* = 6). *indicates a statistically significant difference to control with *p* < 0.05 (Student’s *t* test, *R*).

To assess photosynthesis function, leaf fluorescence was measured *in vivo* on tomato plants previously sprayed one or three times with COS-OGA (62.5 mg/l) or 0.1% Tween 20. Photosynthesis parameters were assessed directly after spraying and 24 h later. COS-OGA did not statistically modify the maximal quantum yield (Fv/Fm) nor the fraction of open reaction centers (qL) at any time (Supplementary Figure 2). Concerning PSII operating efficiency (ΦPSII) and NPQ, the only significant differences between COS-OGA-elicited plants and control plants were recorded 24 h after the first spraying. At this moment, COS-OGA-treated plants underwent a significant reduction in ΦPSII (−8.2%), while NPQ was significantly increased in these plants (+45%; Figure 3).

### Pigment Contents

Pigment contents (chlorophyll *a* and *b*, β-carotene, violaxanthin, and neoxanthin) were evaluated 24 h after the third treatment with COS-OGA (62.5 mg/l) or 0.1% Tween 20.

**FIGURE 4:**
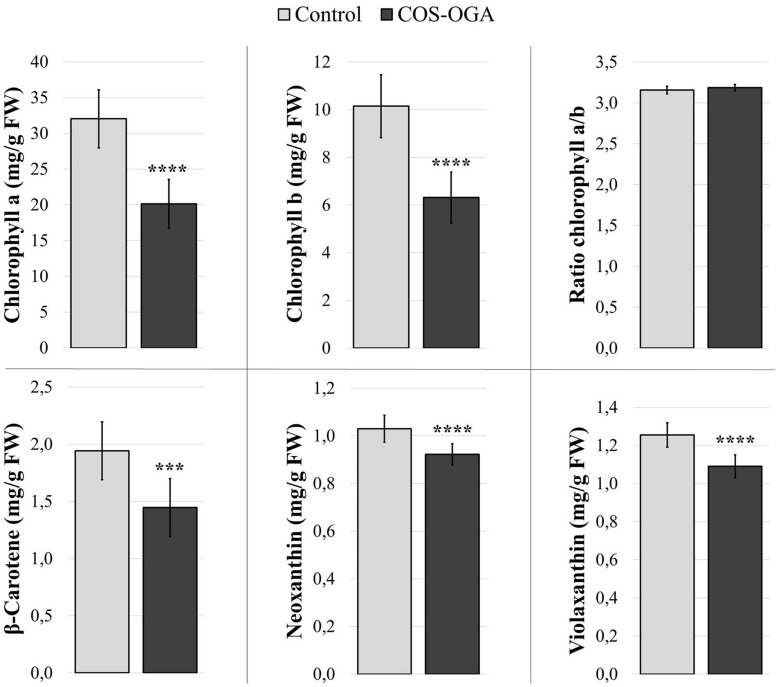
Pigment contents of tomato leaves after three sprayings with chitooligosaccharides–oligogalacturonides (COS-OGA; 62.5 mg/l). Tomato leaves were harvested 24 h after the third treatment. The average pigment contents are expressed as mg/g fresh weigh ± standard deviation (*n* = 12). *** and **** indicate statistically significant differences to the control group with *p* < 0.001 and *p* < 0.0001, respectively, (Wilcoxon test, *R*).

Elicited plants showed a significant decrease in all measured pigments: Chlorophyll *a* and *b* dropped by 37%, while the ratio between chlorophyll *a* and chlorophyll *b* was not statistically different between control and elicited plants. β-Carotene showed a 26% decrease, and neoxanthin and violaxanthin contents were reduced by 10% and 13%, respectively, (Figure 4).

Chlorophyll *a* and *b* contents were also evaluated just before the third treatment with COS-OGA (62.5 mg/l) or 0.1% Tween 20 and 24 h later. Elicited plants showed a significant decrease in chlorophyll *a* and *b* at both moments. The ratio between chlorophyll *a* and chlorophyll *b* was not modified by this third treatment (Supplementary Figure 3).

### Stomatal Conductance

**FIGURE 5:**
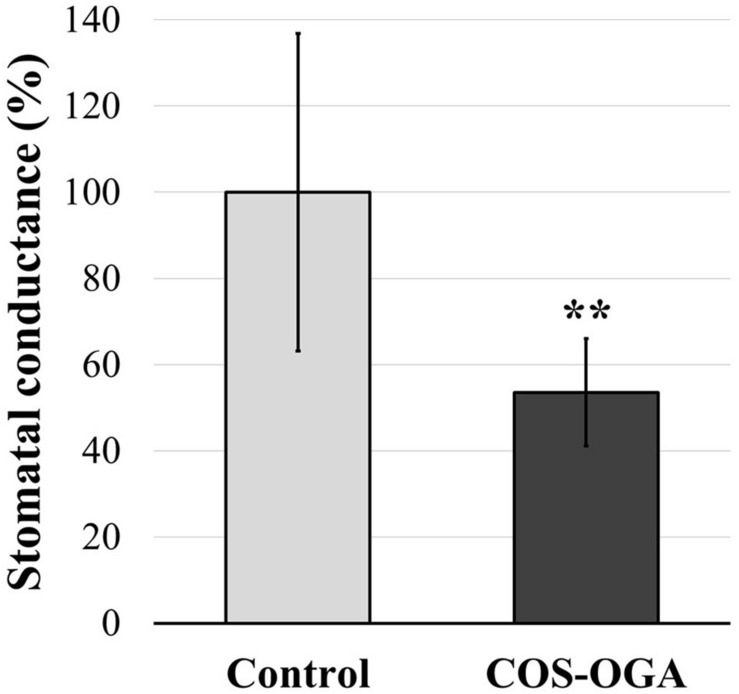
Stomatal conductance of tomato leaves after three sprayings with chitooligosaccharides–oligogalacturonides (COS-OGA; 62.5 mg/l). The average stomatal conductance of the elicited plants is expressed as a percentage of the control ± standard deviation (*n* = 12). **indicates a statistically significant difference to control according to Student’s *t* test (*p* < 0.01, *R*).

Stomatal conductance was evaluated in tomato plants after three treatments with COS-OGA (62.5 mg/l). Tomato leaves showed a significant decrease (−46%) in stomatal conductance 24 h after the third elicitation (Figure 5).

### Fresh and Dry Weights of Tomato After Elicitation

Fresh and dry weights of tomato shoots were evaluated 24 h and 7 days after the third treatment with COS-OGA (62.5 mg/l) or 0.1% Tween 20. Elicitation did not induce any statistically significant difference in tomato dry and fresh weights (Supplementary Figure 4).

### Reduced Nicotinamide Adenine Dinucleotide Phosphate Content

**FIGURE 6:**
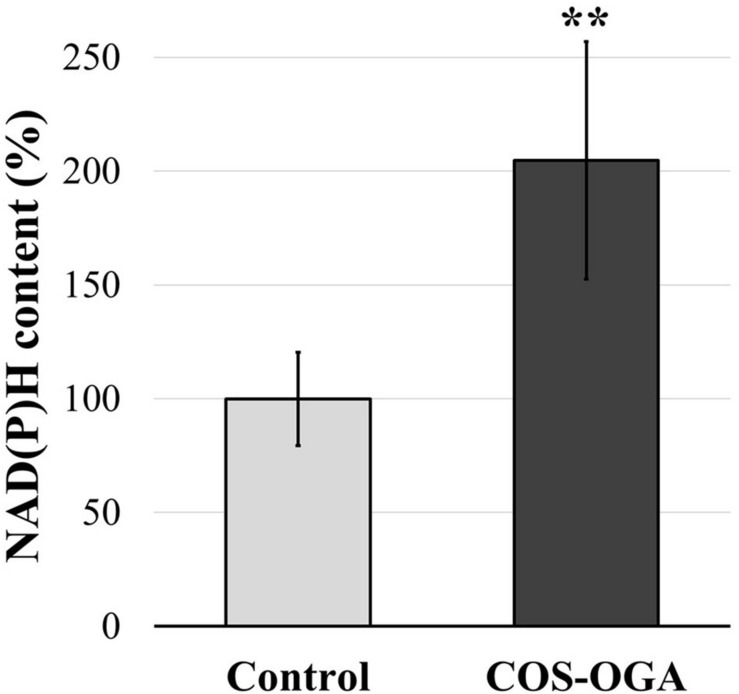
Reduced nicotinamide adenine dinucleotide phosphate [NAD(P)H] content of tomato leaves after three sprayings with chitooligosaccharides–oligogalacturonides (COS-OGA; 62.5 mg/l). Tomato leaves were harvested 24 h after the third treatment. The average NAD(P)H content is expressed as the average percentage of the control ± standard deviation (*n* = 6). **indicates a statistically significant difference to control according to Student’s *t* test (*p* < 0.01, *R*).

Nicotinamide adenine dinucleotide phosphate contents were evaluated in tomato plants 24 h after the third treatment with COS-OGA (62.5 mg/l) or 0.1% Tween 20. Elicited leaves showed a significant increase in NAD(P)H content up to 205% in comparison to the controls (Figure 6).

### Peroxidase Activity

**FIGURE 7:**
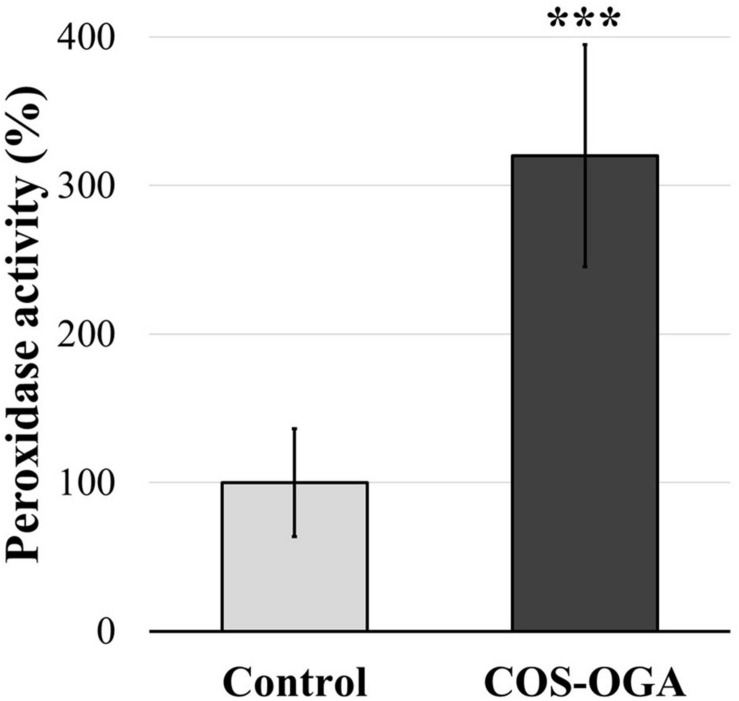
Normalized class III peroxidase activity of tomato leaves after three sprayings with chitooligosaccharides–oligogalacturonides (COS-OGA; 62.5 mg/l). Tomato leaves were harvested 24 h after the third treatment. The average peroxidase activity of the elicited plants is expressed as a percentage of the control ± standard deviation (*n* = 12). ***indicates a statistically significant difference to control according to Student’s *t* test (*p* < 0.001, *R*).

The activity of class III peroxidases was evaluated in tomato plants after three treatments with COS-OGA (62.5 mg/l) or 0.1% Tween 20. Plants tested 24 h after the last COS-OGA elicitation showed a highly significant increase in peroxidase activity (up to 320%) as compared to controls (Figure 7).

### Apoplastic Reactive Oxygen Species Production

**FIGURE 8:**
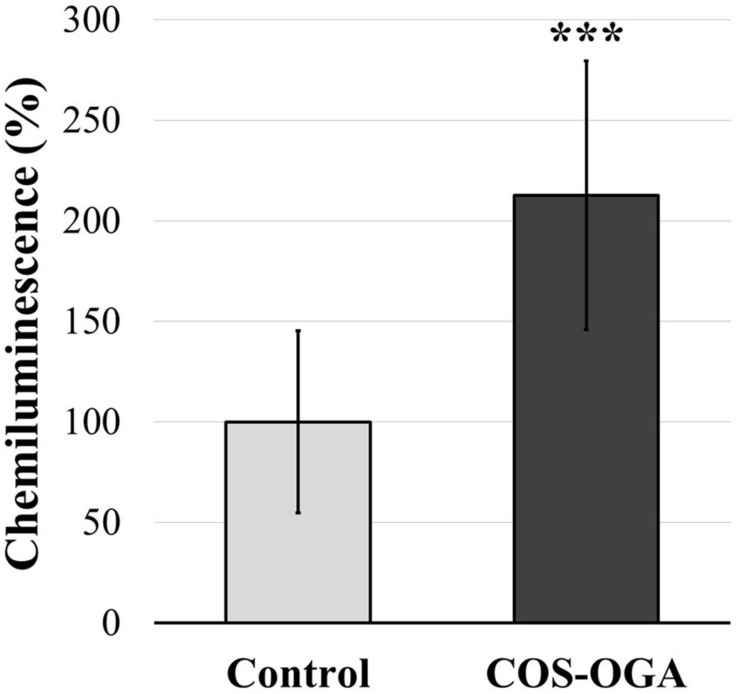
Normalized apoplastic reactive oxygen species (ROS) production in tomato leaf disks. Tomato leaf disks were treated with 0.1% Tween 20 (control) or chitooligosaccharides–oligogalacturonides (COS-OGA; 62.5 mg/l). The average apoplastic ROS production of the COS-OGA-treated leaf disks is expressed as a percentage luminol chemiluminescence emitted in comparison to the control ± standard deviation (*n* = 12). ***indicates a statistically significant difference to control according to Student’s *t* test (*p* < 0.001, *R*).

Apoplastic ROS production was evaluated in tomato leaf disks soaked in COS-OGA (62.5 mg/l) or 0.1% Tween 20 (control). Leaf disks immerged in COS-OGA showed a significant increase in ROS production up to 213% as compared to controls (Figure 8).

## Discussion

Elicitation of plant cells induces a whole array of cytoplasmic and nuclear defense responses, a topic currently subject to particular attention by plant pathologists. However, many if not most studies to date have been done on the dicot model plant *A. thaliana* or on a few other plants but following only one single elicitation. Here, we studied the effect of repeated elicitations of tomato by the COS-OGA elicitor. We treated tomato plants three times with the elicitor and compared their transcriptomes to control plants using RNA-Seq.

When looking at gene transcription in control and elicited plants, the first striking difference observed concerned photosynthesis (Figures 1, 2): elicited plants underwent a massive upregulation of transcripts involved in light reactions and to a lower extent in the Calvin–Benson cycle.

Chloroplasts are the source of metabolic intermediates required for plant growth, including phytohormones, carbohydrates, and amino acids. Chloroplasts are also the production site of several defense compounds including salicylic acid and secondary metabolites (e.g., flavonoids, lignans, and alkaloids), two pathways whose transcription is upregulated following COS-OGA treatment (Figure 1). Previous studies had already pointed out the upregulation of photosynthesis-related genes 24 h after treatment with BTH, chitosan, or the commercial product Romeo^®^, probably to meet metabolic needs for plant defense and/or in response to ROS production (Landi et al., 2017; De Miccolis Angelini et al., 2019). Alternatively, in an article studying the early transcriptomic response of potato leaves treated with chitosan, such an upregulation was interpreted as a way to increase the photosynthetic capacity (Lemke et al., 2020).

The involvement of the chloroplast machinery may also result in a large ROS production due to light-driven electron flow in thylakoids (Foyer, 2018). Singlet oxygen (^1^O_2_) is mainly produced in PSII from chlorophyll P680 when the reduced plastoquinone pool is no longer able to accept electrons (Janku et al., 2019). Even in optimal growth conditions, PSII is prone to photooxidative damages and must be continuously and rapidly repaired, an energy-consuming process referred to as PSII repair cycle (Lu, 2016; Theis and Schroda, 2016; D’Alessandro and Havaux, 2019). To prevent PSII over-excitation and avoid exceeding the PSII repair cycle capacity, photo-protective systems are switched on. Among them, NPQ is a short-term response to excess light that enables the dissipation of chlorophyll excitation energy as heat (Ruban, 2016; Townsend et al., 2018). 1 day after the first COS-OGA treatment, the ΦPSII decrease reflected a lower ability to oxidize Qa probably because the energy captured by the PSII antenna exceeded the electrons needed for CO_2_ assimilation (Baker, 2008). At the same time, the NPQ capacity for energy dissipation increased, a phenomenon often detected upon biotic and abiotic stress (Figure 3; Pérez-Bueno et al., 2019).

However, after three sprayings, elicited plants did not increase NPQ anymore above controls. As suggested by the constant maximum quantum yield, COS-OGA spraying did not induce any photoinhibition, neither directly nor 24 h after the third treatment. Furthermore, the efficiency at which light was absorbed by PSII and used to perform chemistry was not statistically modified (ΦPSII and qL, Figure 3 and Supplementary Figure 2; Kalaji et al., 2014, 2017). Therefore, COS-OGA elicitation probably did not lead to any transient over-reduction of PSII, avoiding ^1^O_2_ generation.

**FIGURE 9:**
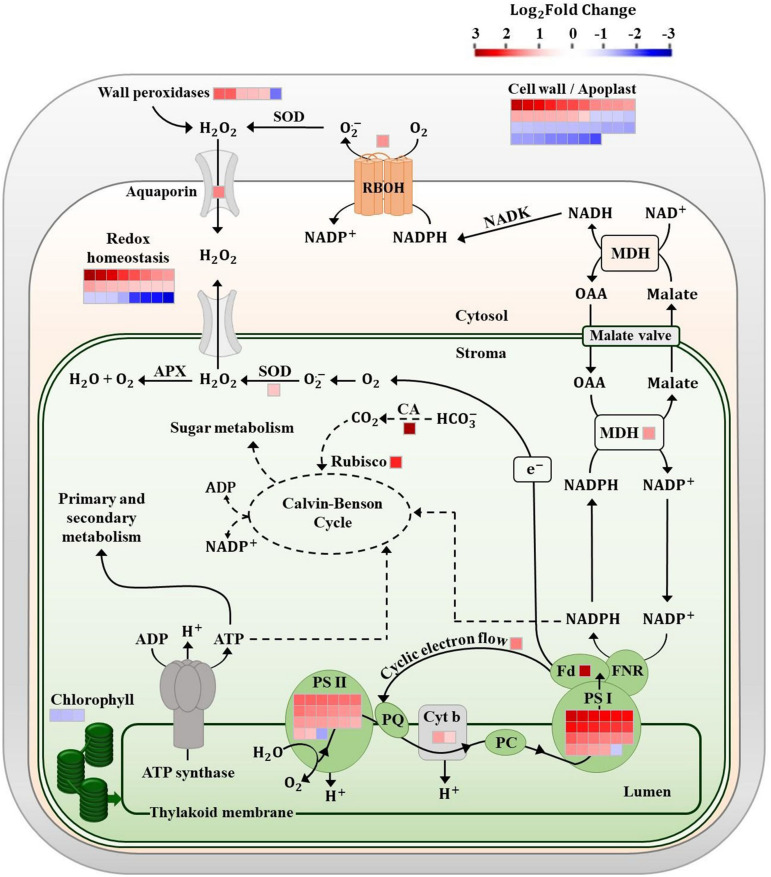
Chloroplast electron chain and redox homeostasis in tomato leaves after three sprayings with chitooligosaccharides–oligogalacturonides (COS-OGA; 62.5 mg/l). The electrons produced by the chloroplast light phase are used for CO_2_ reduction, reduced nicotinamide adenine dinucleotide phosphate (NADPH) production in the cytosol *via* the malate valve, and H_2_O_2_ accumulation. Differentially expressed genes in elicited versus control leaves are represented by squares [false discovery rate (FDR) < 0.05; |log_2_Fold Change| > 1]. Squares are colored in red and blue if they are upregulated and downregulated, respectively. APX, ascorbate peroxidase; CA, carbonic anhydrase; Cyt b, cytochrome b; Fd, ferredoxin; FNR, ferredoxin NADP^+^ reductase; MDH, malate dehydrogenase; NADK, NAD kinase; OAA, oxaloacetic acid; PC, plastocyanin; PQ, plastoquinone; PS, photosystem; and SOD, superoxide dismutase.

The response to COS-OGA spraying clearly differed between the first and the third elicitation, suggesting an acclimation mechanism to maintain optimal photosynthetic efficiency. Besides, all pigment contents were reduced in elicited plants 24 h after the third treatment (Figure 4), which is in agreement with the downregulation of two protochlorophyllide reductases involved in chlorophyll biosynthesis (Figure 9; Bryant et al., 2020). This decrease is not caused by the third treatment given that plants sampled before this spraying already had reduced pigment contents (Supplementary Figure 3). A chlorophyllase was also downregulated, an enzyme whose physiological role is still a matter for debate (Queiroz Zepka et al., 2019). Chlorophyllases were first considered to be involved in the first steps of chlorophyll degradation (Siddiqui et al., 2019; Altuntaş et al., 2020; Liu et al., 2020). Other reports show that these enzymes are not located in the chloroplast and are not involved in chlorophyll degradation during senescence or heat stress (Lin et al., 2014; Hu et al., 2020). Chlorophyllases are also involved in chlorophyll degradation after thylakoid membrane disruption (Kariola et al., 2005; Hu et al., 2015). Nevertheless, even if the chlorophyll content does not influence F_V_/F_M_ measurements, it has an impact on NPQ, qL, and ΦPSII values. Indeed, the fluorescence detected is probably mainly emitted by chloroplasts located near the adaxial leaf surface of the top leaves (Tsuyama et al., 2003; Dinç et al., 2012; Mänd et al., 2013). The parameters measured, especially NPQ, qL, and ΦPSII, do not reflect the photosynthetic capacities of all the chloroplasts present in the leaves (Peguero-Pina et al., 2009), although pigment contents were measured on whole leaves.

Taken together, the decrease in pigment content, the downregulation of chlorophyll-related genes, and the upregulation of a large number of transcripts involved in light reactions point to an acclimation mechanism implemented on a time scale of days rather than on a short-term response like NPQ. Literature describes such long-term acclimation of photosynthetic apparatus to be based on two main mechanisms: the stoichiometry of the different components of the electron chain (PSII, PSI, cytochrome b6/f, plastocyanins, and ATP synthase; Kim et al., 1993; Fan et al., 2007; Puthiyaveetil et al., 2012) on the one hand and modifications of PSII antenna size on the other hand (Wituszyńska et al., 2013). Both mechanisms seem to be linked to each other (Chow et al., 1990; Wituszyńska et al., 2013; Schöttler and Tóth, 2014; Jia et al., 2016; Bielczynski et al., 2020).

Several reports show that a reduction in PSII antenna size results in an increase in plant biomass. As a very small proportion of the energy captured by the antenna is used in photochemistry, reduced antenna size mitigates photoinhibition and improves light penetration deeper into plant tissues and through leaf stages (Melis, 2009; Shin et al., 2016; Gu et al., 2017; Song et al., 2017; Dall’Osto et al., 2019; Takagi et al., 2019; Patil et al., 2020). PSII antenna size reduction is generally coupled to a decrease in Chl *a/b* ratio (Esteban et al., 2015), but in our case, Chl *a/b* ratio was not modified and all measured pigments were affected, whether bound to the light harvesting complex or not (Figure 4). The enrichment of transcripts related especially to PSI but also to PSII and cytochrome b6/f could indicate modifications in their stoichiometry so as to balance the excitation rates and electron transport between the two photosystems (Figure 9).

Photosystem I may also generate ROS: any disequilibrium between ATP and NADPH production and consumption leads to photoinhibition and ROS production (Lima-Melo et al., 2019). Superoxide anions (^1^O_2_) formed from ferredoxin electrons have a low reactivity, and their production acts as an “electron pressure release valve” when the availability of the final electron acceptor NADP^+^ decreases, which happens in case of high light irradiance and/or CO_2_ shortage caused by drought and/or stomatal closure (Takagi et al., 2016). Here, in tomato, stomatal conductance was reduced by 46% after the third COS-OGA treatment (Figure 5). CO_2_ flux reduction due to such a stomatal closure should have depressed the Calvin–Benson cycle, increased the photorespiratory pathway, lowered NADP^+^ availability, and caused excess excitation energy accumulation in the electron transfer chain and PSI permanent reduction (Eisenhut et al., 2017; Khatri and Rathore, 2019).

Surprisingly, despite this stomatal closure, elicited tomato plants did not undergo any fresh/dry weight reduction and the photorespiratory pathway was not differentially expressed, indicating that additional mechanisms might have alleviated the consequences of CO_2_ shortage (Supplementary Figure 4). Several hypotheses can be proposed such as an increased efficiency in CO_2_ diffusion and/or fixation or the use of an alternative carbon source. Concerning CO_2_ diffusion and fixation, the observed increase in transcript abundance of aquaporin, carbonic anhydrase, and Rubisco large chain might to some extent have balanced the observed decrease in stomatal conductance (Figure 9). In C3 plants, carbonic anhydrase facilitates CO_2_ diffusion and accumulation in the stroma and enables the use of HCO_3_^–^ and xylem-transported CO_2_ from roots as a Calvin cycle substrate, especially when intercellular CO_2_ concentration is low (Dąbrowska-Bronk et al., 2016; Floryszak-Wieczorek and Arasimowicz-Jelonek, 2017; Stutz and Hanson, 2019).

In PSI, there is no recycling mechanism analogous to the PSII repair cycle and PSI damages by excess light lead to the complete resynthesis of the supercomplex (Lima-Melo et al., 2019). Hence, several mechanisms prevent PSI photoinhibition and maintain a correct balance of the ATP/NADPH ratio. These include the CEF, the Mehler cycle, the malate valve, the mitochondrial energy dissipation based on alternative oxidase (AOX), and ROS scavenging systems (Selinski and Scheibe, 2019; Sunil et al., 2019; Lu et al., 2020; Sun et al., 2020).

Cyclic electron flow diverts electrons from linear electron flow and cycles them back into the PQ pool, resulting in a substantial energy dissipation as heat but also in a proton gradient and subsequent ATP production. By avoiding NADP^+^ reduction and producing ATP instead, CEF enables a fine balancing between the ATP/NADPH ratio produced by the linear electron flow and what is actually required for primary and secondary metabolism (Yamori and Shikanai, 2016; Yamori et al., 2016). Under steady-state conditions, CEF involvement is minor but this pathway seems to be upregulated following environmental changes or when a high ATP demand is expected (Huang et al., 2018; Murata and Nishiyama, 2018; Fisher et al., 2019; Tan et al., 2020a, b). In elicited plants, the upregulation of a chloroplastic NDH gene indicates a possible increase in CEF in response to COS-OGA (Figure 9; Yamori et al., 2015; Nikkanen et al., 2018).

The chloroplast redox state also regulates the malate-oxaloacetate shuttle that enables excess NADPH equivalents to be exported to the cytosol, mitochondria, or peroxisome (Beeler et al., 2014; Chen et al., 2019). Since membranes are impermeable to NADPH, the conversion of oxaloacetate to malate by NADP-MDH followed by the export of malate to the cytosol removes high-energy electrons from the chloroplast. Thereafter, NAD(P)H may be formed in the cytosol, peroxisome, or mitochondria by the reverse reaction (Kandoi et al., 2018). The increased expression of NADP-MDH induced by COS-OGA (Figure 9) might serve the purpose of exporting to the cytosol the reducing power generated in the chloroplast. Indeed, after the third elicitation, the NAD(P)H cellular content doubled in comparison to the control (Figure 6). The reduced cofactor could then transfer electrons for example to RBOH, providing electrons for antioxidant enzymes, for mitochondrial ATP synthesis, or even for energy dissipation as heat *via* the alternative respiratory pathway of mitochondria (Selinski and Scheibe, 2019; Figure 9).

In any case, the redox homeostasis seems to strongly respond to COS-OGA elicitation. This is further confirmed by the overexpression of ROS scavengers and ROS-related signaling molecules observed in the RNA-Seq (Figure 1). Cytosol plays a major role in the integration of ROS-related signals originating from the apoplast and from all the organelles in order to modulate gene expression in the nucleus: ROS signals must be strong enough to induce a nuclear response but not too strong to avoid toxicity (Mittler, 2017; Noctor et al., 2018). Following COS-OGA elicitation, ROS-related signaling in the cytoplasm could have been amplified by the downregulation of antioxidant species, as reflected by the reduced expression of Glutathione-S-Transferases (GSTs) and other antioxidants such as catalase (Figure 1; Gullner et al., 2018; Gallé et al., 2019). However, ROS-triggered signaling is based on a complex network that is much more sophisticated than just balancing ROS and antioxidant species. Antioxidants not only keep ROS low but they also act as redox-sensitive molecules reacting to homeostasis perturbations (Noctor et al., 2018). The simultaneous upregulation and downregulation of antioxidants in the cytosol following elicitation reflect the complexity of this ROS-antioxidative interplay.

The tomato leaf cell wall, and more generally the apoplast, is another cellular compartment strongly influenced by COS-OGA sprayings, as evidenced by wall transcript enrichment (Figure 2), the increased peroxidase enzymatic activity (Figure 7), and the increase of apoplastic ROS production in leaf disks following COS-OGA treatment (Figure 8). Apoplastic ROS play a major role in biotic stress as they are involved in cell wall modifications and they directly act on invading pathogens. This is particularly relevant in the case of biotrophic agents such as powdery mildews, well-known to develop in the apoplast of plant hosts without breaching their plasma membrane. Even intracellular hyphae and haustoria always remain on the outer side of the host plasma membrane (O’Connell and Panstruga, 2006). Therefore, in the presence of biotrophic pathogens, plants have to unfold their defenses first in cell walls where ROS production plays a major role.

Following PAMP or DAMP perception, RBOHs produce apoplastic ROS *via* the transfer of electrons from NADPH across the plasma membrane. The subsequent membrane depolarization leads to apoplast alkalinization, thereby allowing H_2_O_2_ production by cell wall peroxidases (Podgórska et al., 2017; Figure 7). Class III peroxidases are known to both use hydrogen peroxide to oxidize aromatic compounds and thereby stiffen the cell wall and generate ROS that besides being toxic to invading pathogens can break covalent bonds and soften cell walls (Cosio and Dunand, 2009). Here, the upregulation of RLKs, an RBOH, and a plasma membrane aquaporin gene fits well into a mechanism where COS-OGA detection induces apoplastic ROS production resulting in hydrogen peroxide transfer to the cytosol (Figures 1, 8; Podgórska et al., 2017; Marcec et al., 2019). Besides, extracellular ROS might also induce cytosolic signaling *via* RLKs: CRKs act as ROS sensors through the oxidation of cysteine residues in their extracellular domains and activate signaling cascades such as MAPKs (Bourdais et al., 2015). In parallel, COS-OGA detection by PRRs triggers signaling transduction based on calcium, ROS, and phosphorylation cascades resulting in PTI. This leads to the production of defense-related compounds that include PR proteins, secondary metabolites, and phytohormones (Figure 1). Interestingly, RLKs, RBOHs, MAPKs, calcium, and ROS waves that are instrumental in pathogen-triggered signaling have also been identified as key signals in SAA (Białasek et al., 2017; Morales and Kaiser, 2020).

Here, we show that the response to the COS-OGA elicitor involved the modulation of the electron transport chain in thylakoids and impacted the redox homeostasis of different cell compartments including the apoplast. Therefore, beyond its protective effect against biotic stresses, the elicitation appears to be a major modulator of energy partitioning in plant leaves and repeated COS-OGA treatments most probably induce a long-term acclimation of plants, without any metabolic cost in terms of fresh and dry weights. The precise adaptation mechanisms involved in this acclimation have to be further investigated. In particular, the modifications induced by repeated COS-OGA treatments on the antenna size and on the stoichiometry of the electron chains have to be elucidated. Further experiments are also needed to clarify how the metabolism reacts to stomatal closure and if alternative mechanisms could alleviate CO_2_ shortage. The agricultural significance of such long-term acclimation is important since elicitation could improve protective mechanisms not only against biotic but also abiotic stresses.

## Data Availability Statement

The datasets presented in this study can be found in online repositories. The names of the repository/repositories and accession number(s) can be found below: https://www.ncbi.nlm.nih.gov/, PRJNA645061.

## Author Contributions

SM designed and performed the experiments, analyzed the data, and wrote the manuscript. GVA helped to design the experiments and edited the manuscript. Library preparation and sequencing were performed by VIB Nucleomics Core (https://www.nucleomics.be/). RJ performed RNA-Seq analysis and advised for data analyses. PVC initiated the project, contributed to results interpretation and supervised the writing. All authors contributed to the article and approved the submitted version.

## Conflict of Interest

GVA and PVC are members of FytoFend S.A. The remaining authors declare that the research was conducted in the absence of any commercial or financial relationships that could be construed as a potential conflict of interest.

## Footnotes

1 https://www.nucleomics.be/

2 http://hannonlab.cshl.edu/fastx_toolkit/
