# Supplementary material for: Chloroplast Electron Chain, ROS Production, and Redox Homeostasis Are Modulated by COS-OGA Elicitation in Tomato (Solanum lycopersicum) Leaves
Source: Front Plant Sci. 2020 Dec 14;11:597589. doi: 10.3389/fpls.2020.597589 (PMC7768011; doi:10.3389/fpls.2020.597589)
Supplement: Supplementary file 1 [file Data_Sheet_1.docx]

**Supplementary Figures**


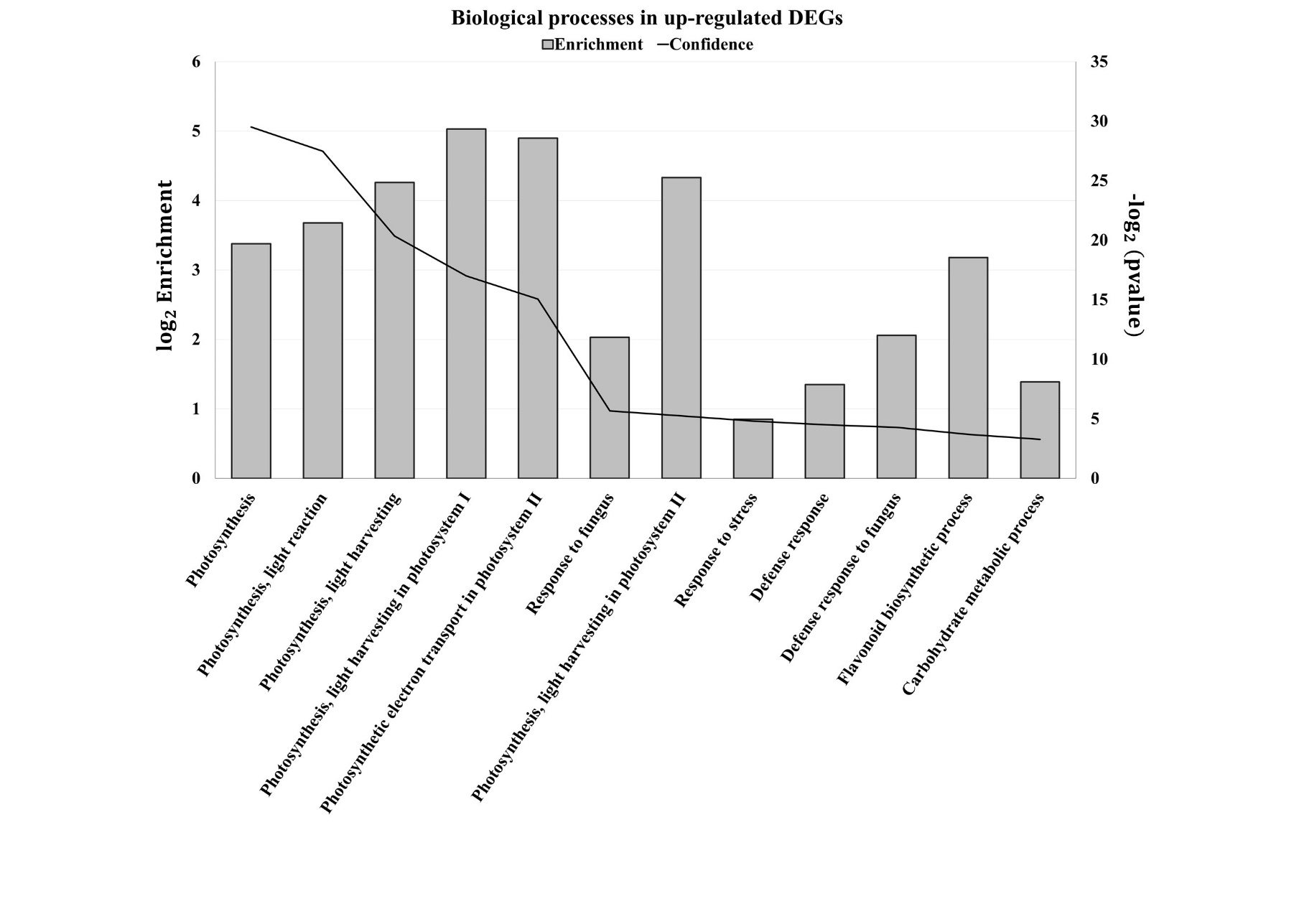


Supplementary Figure 1. Enriched biological pathways (Gene ontology) in up-regulated DEGs of tomato leaves after three sprayings with COS-OGA (62.5 mg/l) (0.1% Tween 20) (based on Plaza project, Dicot 4.5). Tomato leaves *cv.* Moneymaker were spayed seven, two and one day before harvest with either 0.1% Tween (Control) or COS-OGA (62.5 mg/l). Enriched biological pathways (Gene ontology) were identified based on hypergeometric test with Bonferroni correction (p-Values < 0.001). The left axis corresponds to the log_2_ enrichment fold. The right axis represents the – log_2_ (p-Value) represented on the chart by the confidence.


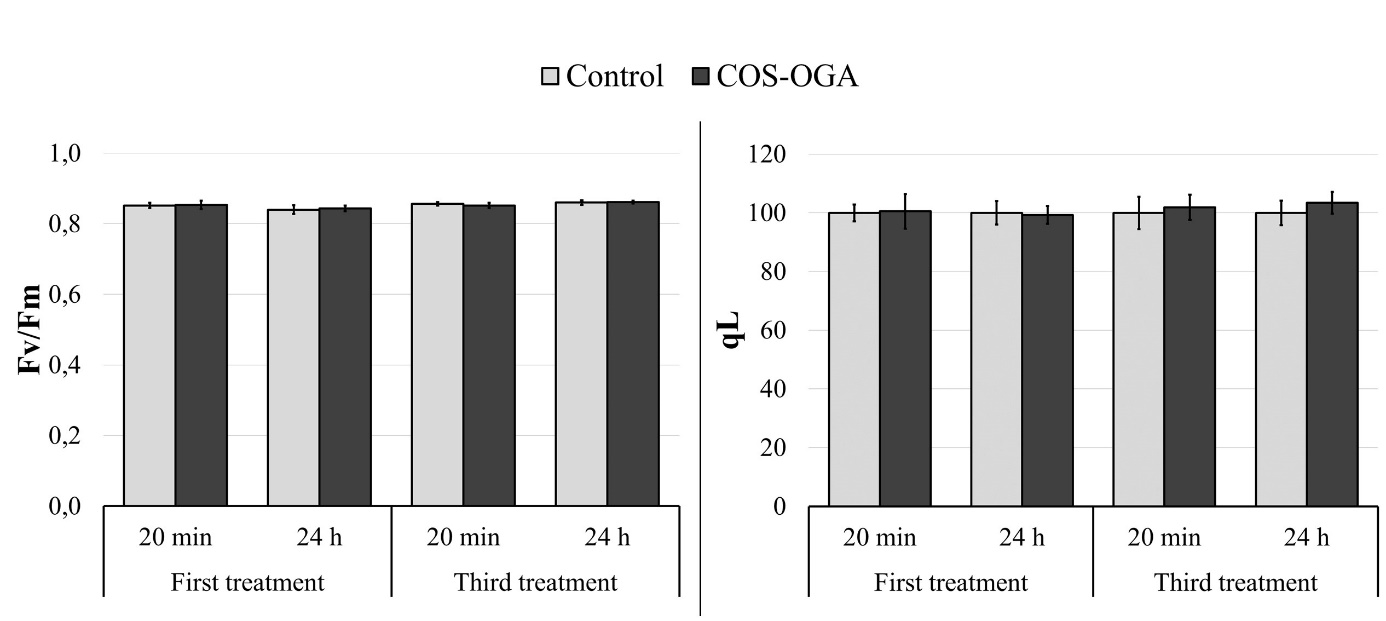


Supplementary Figure 2. Chlorophyll fluorescence parameters of tomato plants 20 minutes and 24 hours after the first and the third sprayings with COS-OGA (62.5 mg/l) or 0.1% Tween 20. The maximum quantum yield (Fv/Fm) and the fraction of oxidized reaction centers (qL) were calculated from fluorescence kinetic data generated during the quenching protocol. These parameters were determined after 20 min of dark adaptation. The average values of fluorescence parameters are expressed as a percentage of the control ± standard deviation (n = 6). No statistically significant differences was found using Student’s t tests.


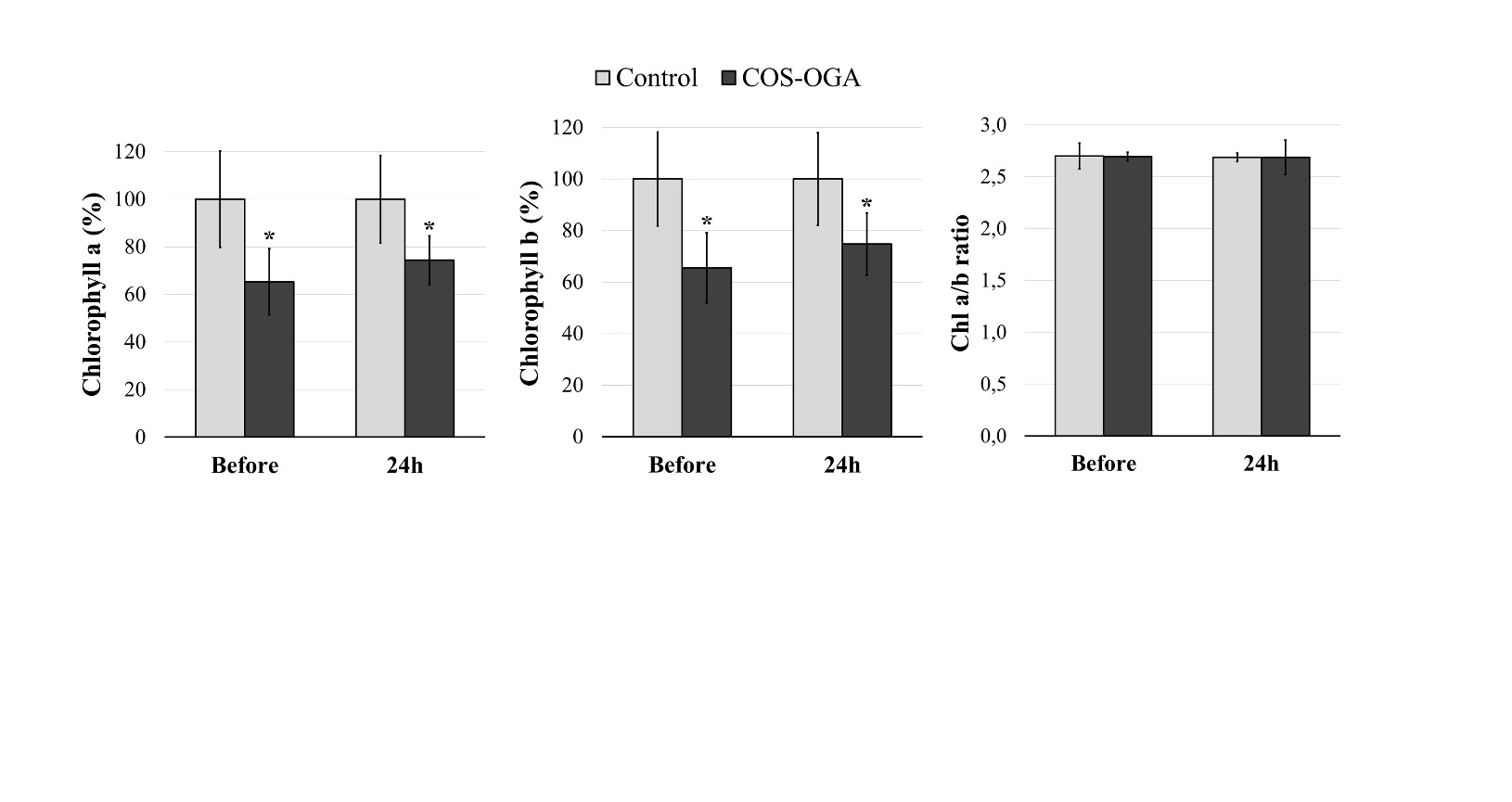


Supplementary Figure 3. Pigment contents of tomato leaves after two or three spraying with COS-OGA (62.5 mg/l) or 0.1% Tween 20. Tomato leaves were harvested just before the third treatment and 24 hours after the third treatment. Pigment contents are expressed as the average percentage of the control ± standard deviation (n = 6).* indicates statistically significant difference to the control group according to Wilcoxon test (p < 0.05, R).


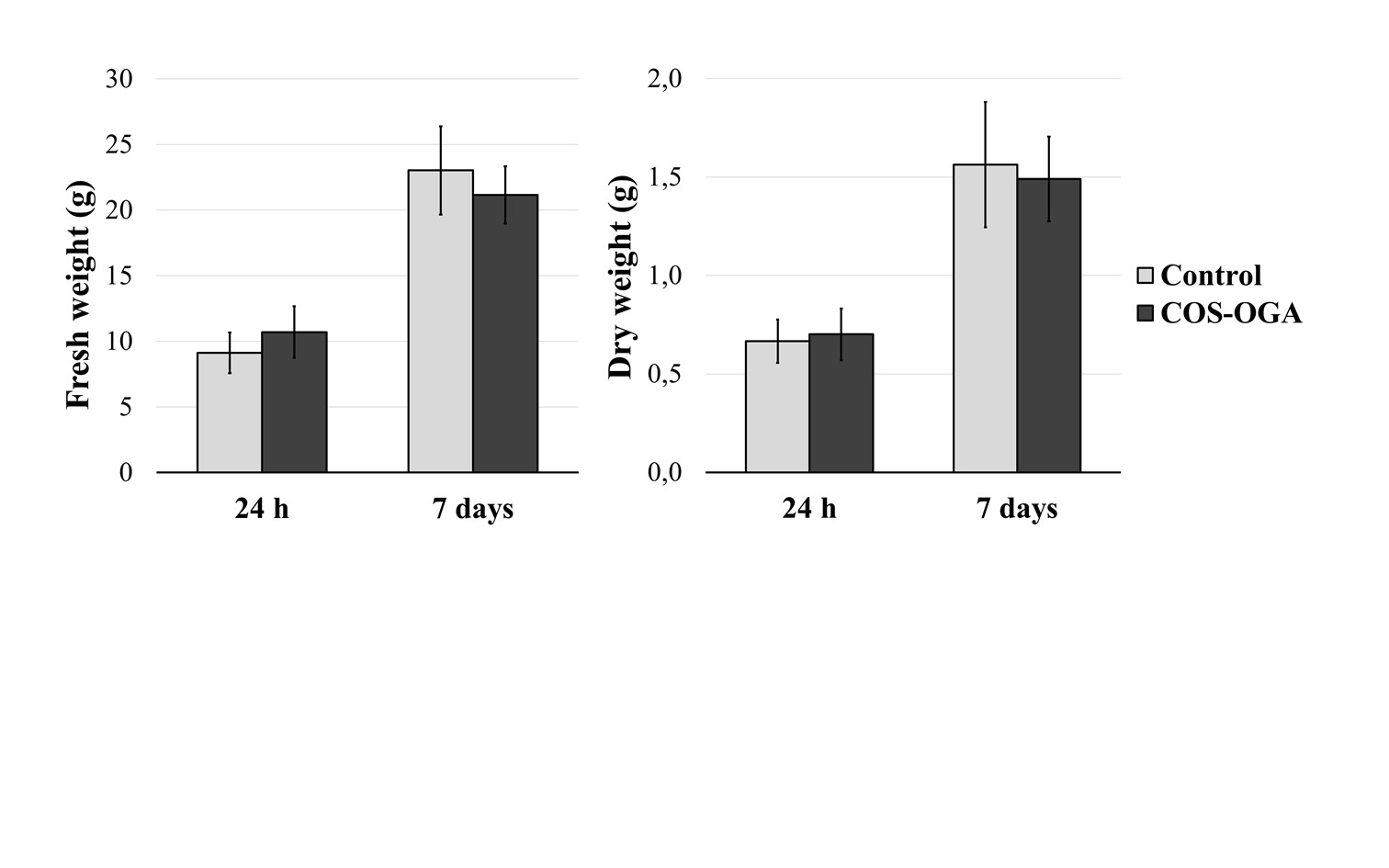


Supplementary Figure 4. Fresh and dry weights of tomato shoots 24 hours and 7 days after the third spraying with COS-OGA. Tomato leaves *cv.* Moneymaker were spayed seven, two and one day before harvest with either 0.1% Tween (Control) or COS-OGA (62.5 mg/l). The average fresh and dry weights are expressed in grams ± standard deviation (n = 10). No statistically significant differences was found using Student’s t tests.
